# Validation of an AI Model for Automated Detection of Alveolar Bone Changes Post-orthodontics Using Cone-Beam Computed Tomography

**DOI:** 10.7759/cureus.94809

**Published:** 2025-10-17

**Authors:** Shibam Bardhan, Gaurav Nagar, Kavya Adapala, Mohsin A Wani, Apoorv Tomer, Sunegha Kundal, Manish Sharma

**Affiliations:** 1 Department of Orthodontics, Teerthanker Mahaveer Dental College and Research Centre, Moradabad, IND; 2 Department of Periodontics, Manav Rachna Dental College, Faridabad, IND; 3 Department of Periodontics, Bharati Vidyapeeth (Deemed to be University) Dental College and Hospital, Sangli, IND; 4 Department of Orthodontics, Desh Bhagat Dental College and Hospital, Mandi Gobindgarh, IND; 5 Department of Orthodontics, Kanti Devi Dental College and Hospital, Mathura, IND; 6 Department of Oral Pathology, Jawahar Medical Foundations Annasaheb Chudaman Patil Dental College, Dhule, IND

**Keywords:** alveolar bone, artificial intelligence, orthodontics treatment, precision, predictors

## Abstract

Introduction: With recent advances in artificial intelligence (AI), image-based tools are increasingly being explored to assist clinicians in detecting changes in alveolar bone levels following orthodontic treatment. This retrospective, cross-sectional study aimed to develop and validate an automated AI model for quantifying alveolar bone levels using cone-beam computed tomography (CBCT) images from patients who underwent fixed orthodontic therapy, with the objective of curating a robust dataset, training AI models for landmark detection, evaluating accuracy against manual annotations, and assessing clinical applicability.

Materials and methods: At the Department of Orthodontics, 1,200 CBCT images from 200 patients (104 (52%) males, 96 (48%) females; mean age 23.6 ± 2.37 years) yielded 3,600 interdental sites. Images covering the maxillary and mandibular segments were preprocessed using median filtering and contrast-limited adaptive histogram equalization. The AI pipeline employed You Only Look Once, version 8 (YOLOv8; Ultralytics, Frederick, MD, USA) for tooth segmentation (720 training, 240 validation, and 240 test images) and a mask region-based convolutional neural network (Mask R-CNN) for generating tooth, bone, and crown masks. Custom algorithms were developed to localize the cementoenamel junction (CEJ) and alveolar bone crest (ALC). A random forest regression model was then applied to identify predictors of alveolar bone loss.

Results: The cohort of 200 patients (104 males, 52%; 96 females, 48%; mean age 23.6 ± 2.37 years) showed alveolar bone loss in 124 patients (62%). The mean periodontal index was 1.90 ± 0.57 mm, and the mean orthodontic treatment duration was 26.72 ± 4.41 months. The mean ALC levels were 1.35 ± 0.65 mm in the anterior region and 1.47 ± 0.62 mm in the posterior region. YOLOv8 achieved a mean average precision (mAP) of 0.941 at an intersection-over-union (IoU) threshold of 0.5 (mAP50) and a tooth detection accuracy of 95.44% at complete IoU. Post-augmentation, Mask R-CNN yielded accuracies of 92.28% (tooth mask), 94.15% (bone mask), and 94.11% (crown mask). CEJ precision ranged from 85.3% to 94.5%, and ALC precision ranged from 82.8% to 95.3%, with a root mean square error of 0.023-0.071. Random forest regression identified treatment duration (importance score: 0.321) as the primary predictor of bone loss.

Conclusion: This AI-driven pipeline offers an efficient and accurate tool for periodontal monitoring, enabling early detection of bone changes and supporting personalized post-orthodontic care. Future prospective studies with diverse cohorts are required to enhance clinical integration.

## Introduction

Orthodontic treatments, particularly fixed appliances, have revolutionized the correction of malocclusions and dental alignments, improving both aesthetics and function in millions of patients worldwide. However, these interventions can inadvertently affect periodontal structures, leading to alveolar bone resorption, gingival recession, and potential long-term complications if not monitored effectively [1]. Periodontal health is crucial post-treatment, as bone loss around teeth can compromise stability and increase susceptibility to diseases. Traditional assessment methods, such as clinical probing and two-dimensional radiography, often fail to provide comprehensive three-dimensional insights into bone morphology, especially in complex post-orthodontic scenarios [2].

Cone-beam computed tomography (CBCT) has emerged as the gold standard for detailed imaging in dentistry, offering high-resolution, volumetric views of alveolar bone and dental roots with reduced radiation compared with conventional CT [3]. CBCT enables the precise evaluation of key landmarks, such as the cementoenamel junction (CEJ) and alveolar bone crest (ALC), which are essential for quantifying bone height and detecting early signs of periodontal deterioration [3,4]. Despite its advantages, manual segmentation and measurement of CBCT scans are labor-intensive, subject to interobserver variability, and time-consuming, limiting their accessibility in routine clinical practice.

The integration of artificial intelligence (AI) into dental imaging represents a transformative shift, automating complex analyses and enhancing diagnostic accuracy [5]. Recent advancements have demonstrated AI's efficacy in segmenting teeth and alveolar bone from CBCT data, facilitating non-invasive periodontal assessments, and supporting personalized treatment planning [6]. AI-driven tools have also shown promise in orthodontic contexts, such as predicting root movements and bone responses during therapy. By leveraging machine learning algorithms, these systems can process large datasets retrospectively, identify patterns that elude human evaluation, and improve outcomes in post-treatment monitoring [6,7].

This retrospective cross-sectional study used AI methodologies to quantitatively assess alveolar bone levels from CBCT images of patients who had undergone fixed orthodontic therapy. This study aimed to develop and validate an automated pipeline for precise localization of the CEJ and ALC, thereby enabling accessible and efficient monitoring of alveolar bone health following orthodontic treatment. The main objective was to develop and validate an AI-based pipeline capable of automatically localizing the CEJ and ALC on post-orthodontic CBCT images to enable precise quantitative assessment of alveolar bone levels. The secondary objective was to identify clinical and treatment-related predictors of alveolar bone loss based on the AI-derived measurements.

## Materials and methods

This retrospective cross-sectional analysis was carried out in the Department of Orthodontics, Teerthanker Mahaveer Dental College and Research Centre, Moradabad, India. The study used AI methodologies to quantitatively evaluate alveolar bone levels using radiographic imaging in patients who had completed fixed orthodontic therapy. The study protocol underwent rigorous ethical review and received formal approval from the Institutional Ethical Committee (TMDCRC/IEC/24-25/PPD31), guaranteeing compliance with international standards for research on human subjects, including data anonymization, informed consent waivers for retrospective data, and secure storage protocols to protect patient privacy.

Data curation and preprocessing

This study was based on a meticulously curated database from the Department of Orthodontics. This repository encompasses the clinical records of at least 200 patients, all of whom had undergone fixed orthodontic therapy during 2020-2024 and had presented with pre- and post-treatment CBCT scans. From this cohort of CBCT scans, 1200 radiographic images (six from each case) were extracted, representing a diverse cross-section of adult patients aged 18-45 years with balanced sex distribution and varying malocclusion severities to ensure generalizability.

**Figure 1:**
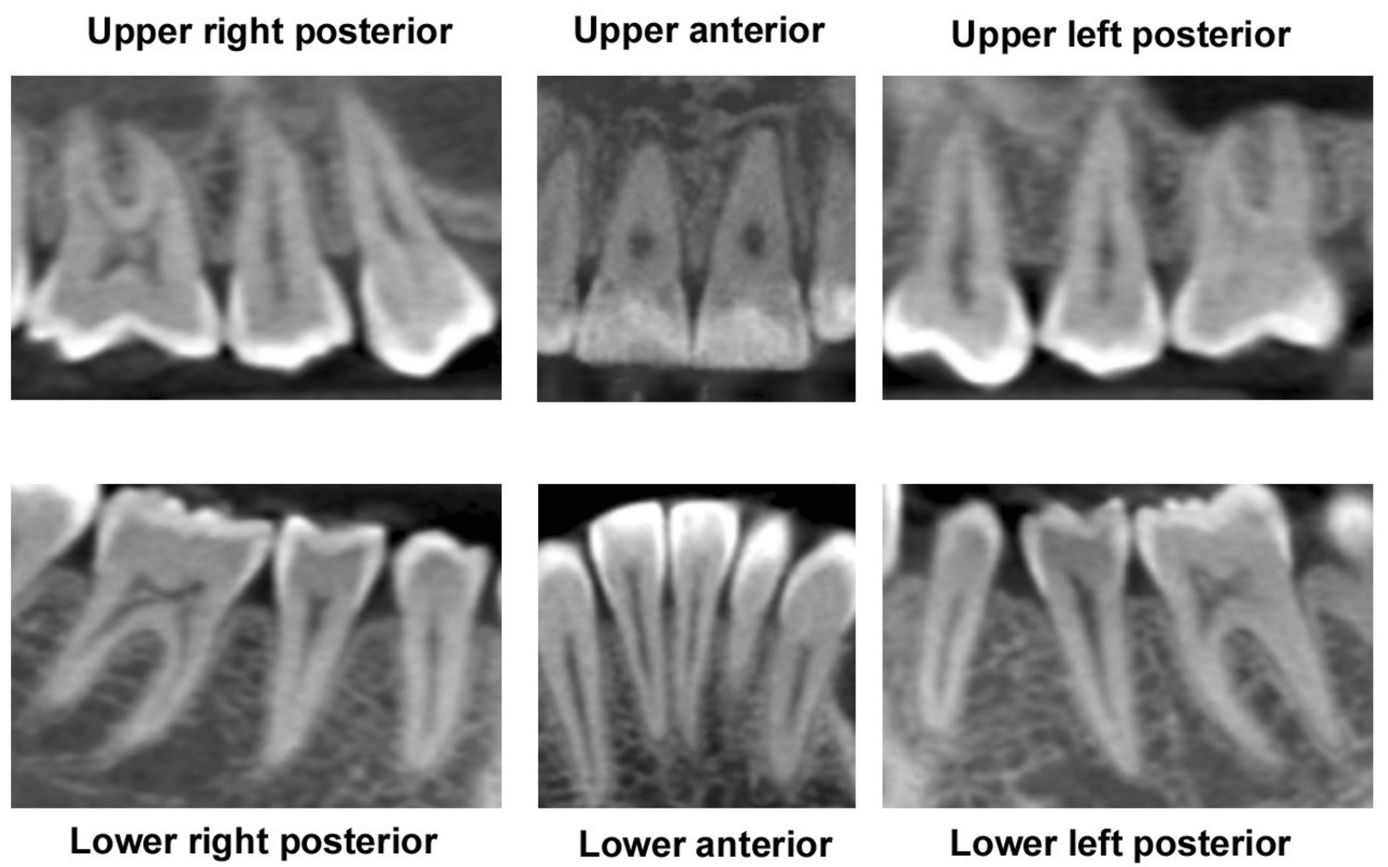
Six radiographic images from a pre-treatment cone-beam computed tomographic (CBCT) scan of the patient. Original CBCT images of a patient from the study.

To standardize the anatomical coverage, six targeted images were derived from each patient's full CBCT volume: maxillary anterior segment (covering incisors and canines), mandibular anterior segment, maxillary right posterior (premolars and molars), mandibular right posterior, maxillary left posterior, and mandibular left posterior (Figure 1). This segmental approach allowed for a comprehensive evaluation across the dentition, capturing region-specific variations in bone morphology influenced by orthodontic forces, such as differential resorption in the anterior versus posterior areas.

Each image focused on three interdental sites (for four included teeth), yielding 3,600 annotated sites as core analytical units. This sample size (n = 3,600) was predetermined through power calculations, assuming a 10% prevalence of clinically significant bone loss and aiming for 95% confidence intervals with a 5% margin of error. This provided ample data for robust AI training while accommodating anatomical diversity, including tooth types (incisors to molars), root configurations, and ethnic variations in periodontal biotypes. Prior to AI processing, all images were preprocessed to enhance the quality and mitigate artifacts inherent to CBCT, such as scatter noise from metallic restorations or beam hardening. A median filter (3 × 3 kernel) was first applied to reduce the Gaussian and salt-and-pepper noise, preserving the edge details essential for landmark delineation. Subsequently, contrast-limited adaptive histogram equalization (CLAHE) was implemented using OpenCV within Python 3.11.9 (Python Software Foundation, Wilmington, DE, USA), with clip limits of 2.0, and tile sizes of 8 × 8 pixels. CLAHE was selected over global histogram equalization for its ability to amplify local contrasts in low-light regions (such as root apices) without overexposing brighter areas (such as enamel), thus sharpening tooth contours, root outlines, and alveolar margins. This step not only improved the segmentation fidelity but also reduced the computational load by standardizing the voxel intensities to a 16-bit grayscale range. Preprocessed images were stored in digital imaging and communications in medicine (DICOM) format, ensuring interoperability with downstream AI frameworks.

AI-driven segmentation and analysis pipeline

The analytical workflow was unfolded into two sequential interdependent stages: coarse tooth instance segmentation, followed by fine-grained mask-based landmark localization. This modular design leveraged the speed of You Only Look Once, version 8 (YOLOv8, Ultralytics, Frederick, MD, USA) for detection, and the precision of the mask region-based convolutional neural network (Mask R-CNN) for segmentation, creating an automated, scalable workflow for clinical use.

Stage 1: Tooth Instance Segmentation using YOLOv8

**Figure 2:**
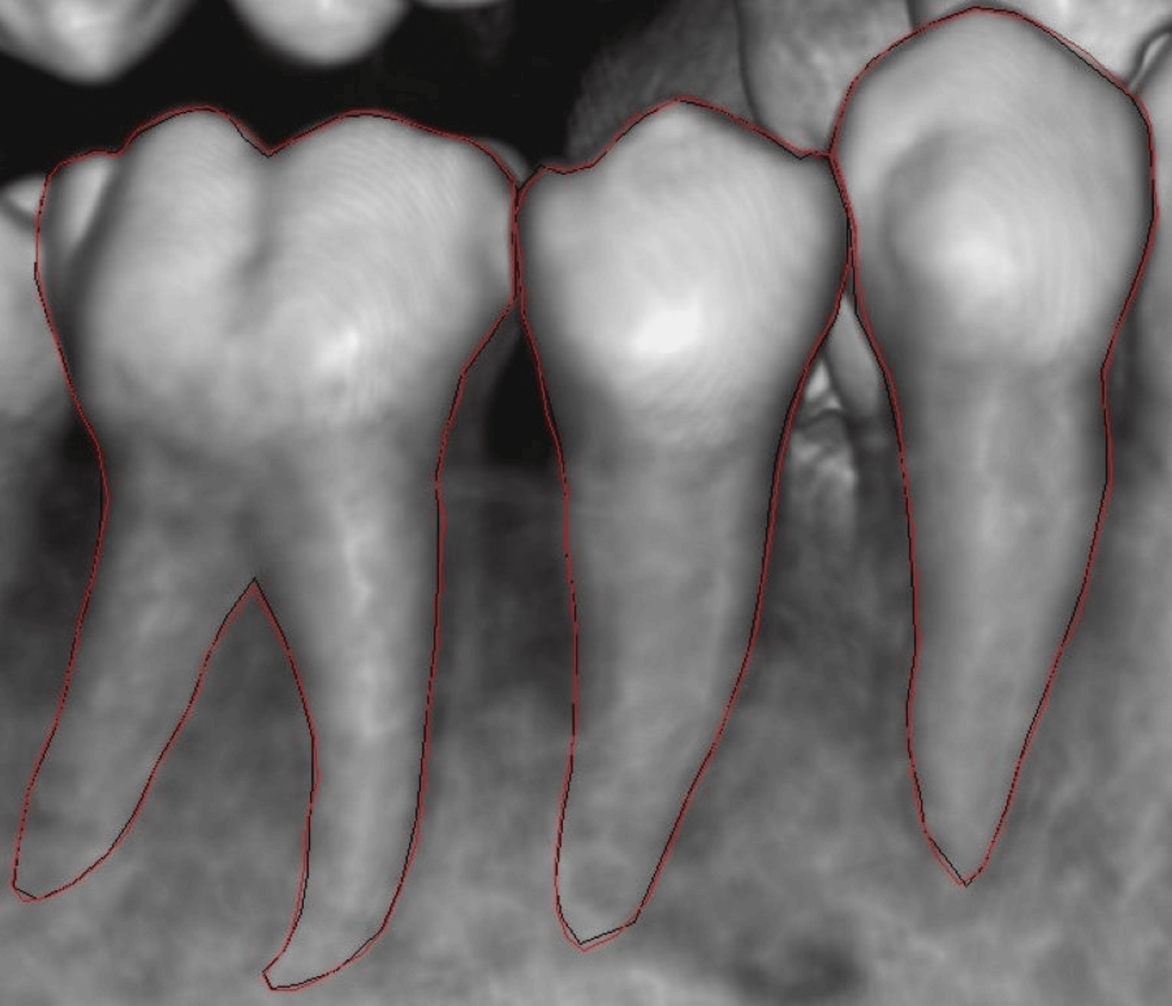
Tooth instance segmentation from a cone-beam computed tomography (CBCT) scan using You Only Look Once, version 8 (YOLOv8) software. Original image derived from the patient’s record.

The pipeline began by isolating individual teeth from the cluttered radiographic field, which is a prerequisite for site-specific analysis. For this, we employed YOLOv8, a state-of-the-art single-stage object detector renowned for its balance of speed (up to 80 FPS on consumer GPUs) and accuracy (mean average precision as mAP@50 > 0.95 on common objects in context as COCO benchmarks). YOLOv8’s anchor-free architecture and cross-stage partial darknet (CSPDarknet) backbone enabled real-time inference, which is ideal for processing high-resolution CBCT slices (512 × 512 pixels) (Figure 2).

A bespoke dataset was assembled by annotating 80% of the preprocessed images (960 images, ~2,880 sites) using the Roboflow annotation tool (Roboflow, Des Moines, IA, USA), which facilitates collaborative polygonal labeling via its web-based interface and version control. Annotators and calibrated orthodontists delineated each tooth with bounding polygons, excluding extraneous structures such as the gingiva or lamina dura. To combat overfitting and augment dataset diversity, critical given the variability in orthodontic-induced remodeling, we applied geometric transformations: random horizontal/vertical flips (probability 0.5), 90-degree rotations (0°, 90°, 180°, 270°), and brightness/contrast jittering (±20%). This expanded the effective dataset by 4×, simulating real-world scan orientations and lighting variances.

The original dataset consisted of records from 200 patients, yielding 1,200 radiographic images derived from CBCT scans. This dataset is divided into three subsets: training, validation, and testing. The training subset included 120 patients, contributing 720 images (with each patient providing six standardized images representing different anatomical regions). The validation subset comprised 40 patients, resulting in 240 images, and the test subset also included 40 patients, contributing an additional 240 images. To enhance the generalization of the model and mitigate overfitting, data augmentation was applied, expanding the dataset to 600 patients' worth of data. The augmented dataset was similarly partitioned: the training subset included 360 patients, yielding 2,160 images; the validation subset consisted of 120 patients, contributing 720 images; and the test subset remained at 40 patients, providing 240 images, as augmentation was not applied to the test set to ensure unbiased evaluation (Table 1).

**Table 1:** Distribution of the dataset. n: number of cases; N: number of radiographic images obtained from case.

Dataset	Training n (N)	Validation n (N)	Test n (N)
Original dataset (n = 200)	120 (720)	40 (240)	40 (240)
Augmentation dataset (n = 600)	360 (2160)	120 (720)	40 (240)

The training proceeded on a custom YOLOv8n (nano-variant) model with a composite loss function integrating binary cross-entropy for classification, complete intersection over union (CIoU) for bounding box regression, and objectness loss for confidence scoring. Hyperparameters were optimized via grid search: initial learning rate 0.01 (decayed by 0.1 at epochs 80/100), batch size 16, 150 epochs, and adaptive moment estimation with weight decay (AdamW) optimizer with weight decay 0.0005. Early stopping halted training if the validation mAP plateaued for 10 epochs. The final model achieved 0.97 mAP@0.5 on validation, with inference times <50 milliseconds per image. Non-assessable teeth, those with obscured CEJs due to artifacts, or featuring implants/crowns, were auto-flagged and excluded via YOLOv8's multi-class head (classes: 'tooth,’ ‘implant,’ ‘artifact'), ensuring pipeline integrity.

Stage 2: Anatomical Landmark Localization using Mask R-CNN

Refined tooth crops from Stage 1 were then processed for pixel-level segmentation using Mask R-CNN, implemented via the Detectron2 framework (Detectron2, Menlo Park, CA, USA; developed by Meta AI Research). Mask R-CNN extends Faster R-CNN with a mask prediction branch, excelling in instance segmentation (average precision @ 0.5:0.95 > 0.40 on COCO). We configured it with a Residual Network of 50 layers and a feature pyramid network (ResNet-50-FPN) backbone for multiscale feature fusion, balancing the depth and efficiency of our hardware.

Training data augmentation included vertical flips (doubling sets to approximately 5,760 samples) and minor scaling (±10%) to handle variations in the root angulation. A key refinement retained only the largest connected component in the tooth mask (via OpenCV’s connected components), discarding fragments from adjacent teeth and focusing analysis on solitary structures.

**Figure 3:**
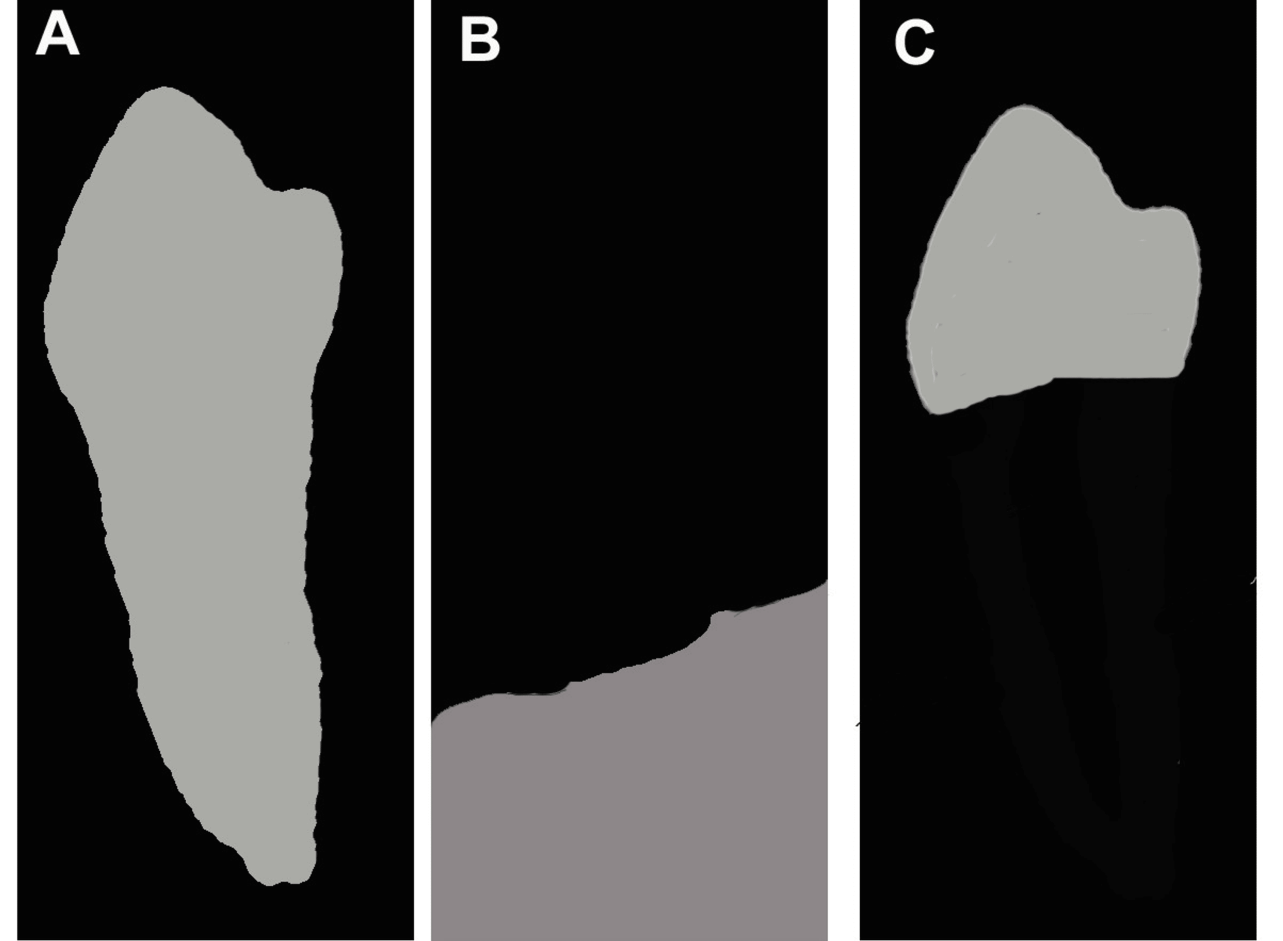
The three types of masks used in this study are (A) tooth annotation mask, (B) bone annotation mask, and (C) crown annotation mask. Original images derived from the patient’s record.

The model outputs three binary masks per tooth: tooth mask (enamel, dentin, and pulp), bone mask (alveolar trabeculae), and crown mask (supragingival enamel) (Figure 3). Annotations for training mirrored Stage 1 but added pixel-wise labels using Roboflow’s polygon-to-mask conversion. We fine-tuned a COCO-pretrained Mask R-CNN checkpoint for 50 epochs (learning rate 0.00025, batch size 8) on PyTorch 2.4.0 (Meta AI Research, Menlo Park, CA, USA), achieving a mask average precision of 0.85. Segmentation leveraged region of interest alignment (RoIAlign) for precise boundary alignment, mitigating sub-pixel errors in CBCT's 0.2-0.4 mm resolution.

For the original dataset, the training subset mirrors the YOLOv8 configuration with 120 patients contributing 720 images (120 × 6), and the validation subset includes 40 patients, providing 240 images (40 × 6), totaling 960 images. The augmented dataset for this model doubles the original data, with the training subset expanding to 240 patients, yielding 1,440 images (240 × 6), and the validation subset increasing to 80 patients, contributing 480 images (80 × 6), totaling 1,920 images. This 2× augmentation factor is lower than YOLOv8’s 3× increase, likely because Mask R-CNN operates on single-tooth images, requiring less variability.

Algorithmic localization of ALC and CEJ

**Figure 4:**
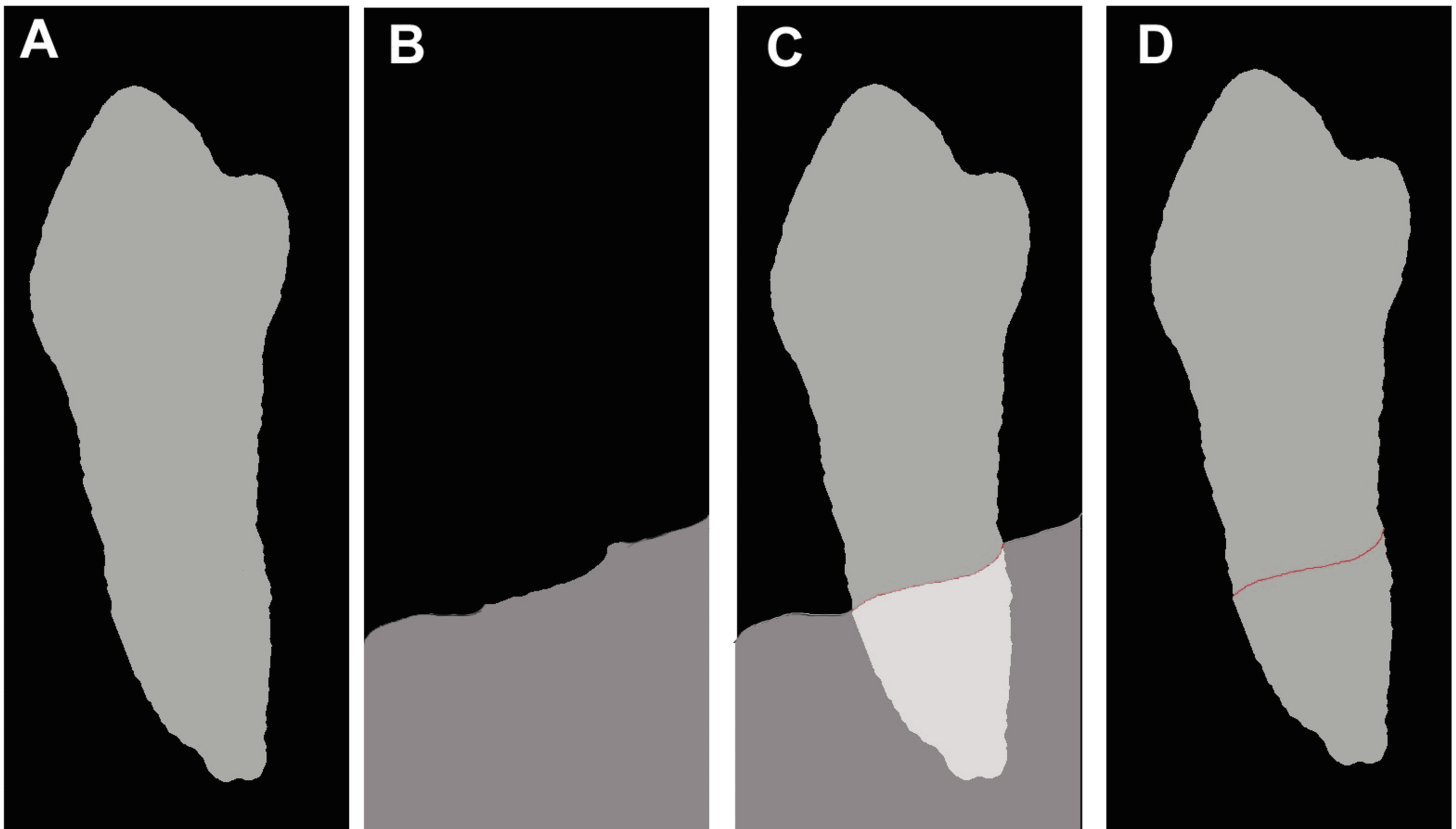
Mask processing in the alveolar bone crest (ALC) level localization algorithm (A) Tooth mask without new value, (B) Bone mask, (C) Overlay, (D) ALC level localization (red line). Original images derived from the patient’s record.

Post-segmentation deterministic algorithms refine landmark detection on mask overlays by bypassing probabilistic deep learning for interpretability. For ALC localization, the tooth mask was morphologically eroded (3 × 3 structures) and overlaid with a bone mask. A 5 × 5 sliding window scanned the composite, identifying the crest as the uppermost y-coordinate, where ≥50% of the window pixels overlapped the tooth root and bone domains (Figure 4). This kernel size approximated the physiological crest widths (1 - 2 mm), yielding a sub-millimeter precision (mean error <0.3 mm).

**Figure 5:**
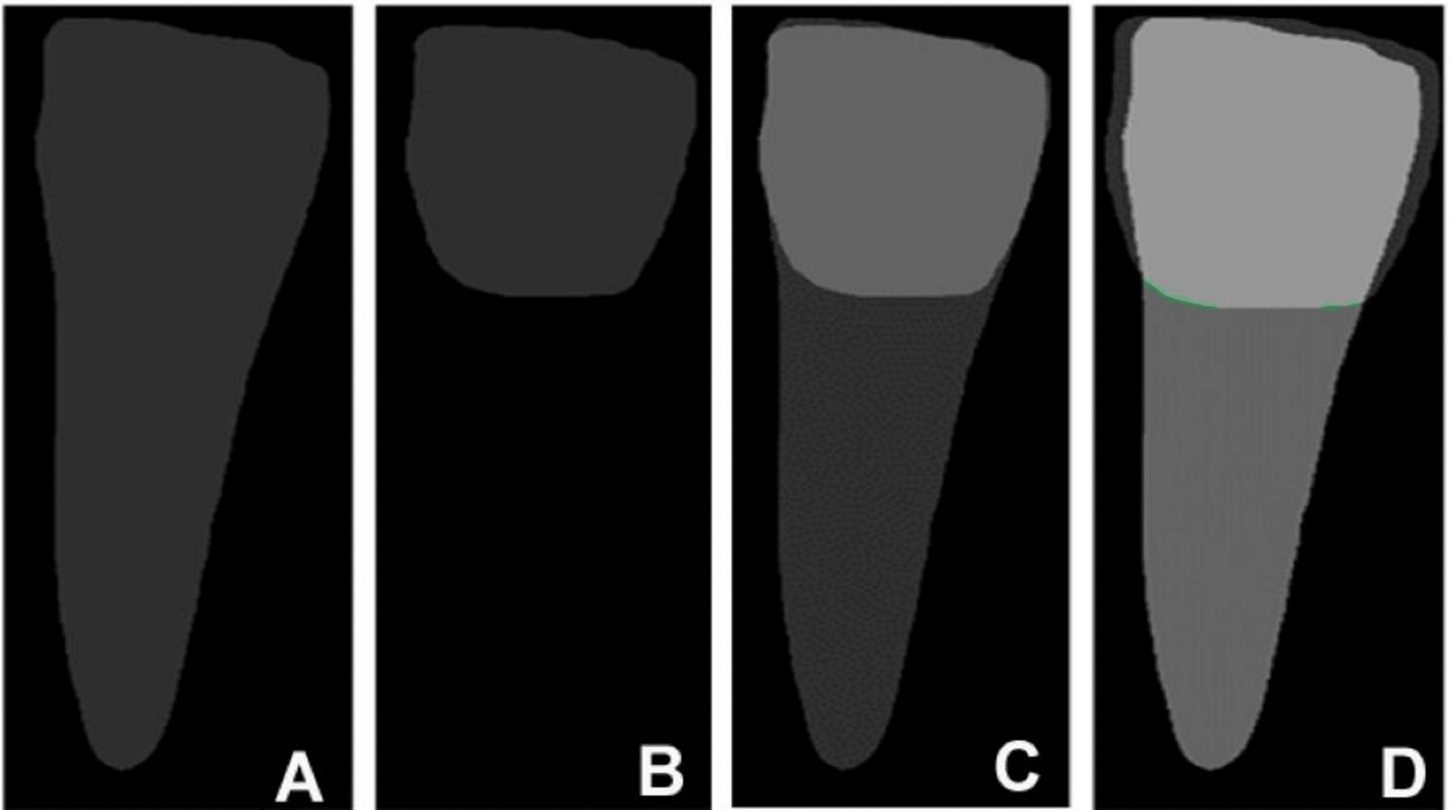
Mask processing in the cementoenamel junction (CEJ) level localization algorithm (A) Tooth mask, (B) Crown mask without dilation, (C) Overlay with dilation, (D) CEJ level localization. Original images derived from the patient’s record.

CEJ localization addresses enamel-root transitions more precisely. The crown mask underwent dilation (5 × 5 disk kernel) to buffer the segmentation noise, and then intersected with the tooth mask. The CEJ was pinpointed at the lowest intersection contour along the mesial/distal aspects and extrapolated linearly for the vertical distance computation (Figure 5). Dilation compensated for ~10-20-pixel inaccuracies in crown segmentation, elevating accuracy from 0.72 to 0.94 (vs. manual). Bone loss was quantified as the vertical CEJ-ALC distance (mm), with thresholds of >2 mm flagging pathology.

The computational pipeline was executed on a system equipped with an advanced microdevice Ryzen 7 3700X central processing unit, an NVIDIA GeForce RTX 3070 graphics processing unit (accelerated using Compute Unified Device Architecture version 12.1, NVIDIA Corp., California, USA), and 32 gigabytes of random-access memory. The implementation was scripted using Python version 3.11.9 (Python Software Foundation, Wilmington, Delaware, USA) (Table 2).

**Table 2:** Computer specifications used in model analysis.

Hardware	Version	Manufacturer
Central Processing Unit (CPU)	AMD Ryzen 7 3700X	Intel, California, United States
Graphics Processing Unit (GPU)	NVIDIA GeForce RTX 3070 *G	NVIDIA Corporation, California, USA
Dynamic Random-Access Memory (DRAM)	32 GB	ADATA, New Taipei City, Taiwan
Software platform	Version	Manufacturer
Python	3.11.9	Python Software Foundation Wilmington, Delaware, USA
PyTorch	2.4.0	Meta Platforms, Inc., California, USA
Anaconda	24.1.2	Anaconda, Inc., Texas, USA
Compute Unified Device Architecture (CUDA)	12.1	NVIDIA Corporation, California, USA

Statistical analysis

Manual annotations by a blinded periodontist served as the ground truth and were exported to Microsoft Excel (Microsoft Corporation, Redmond, WA, USA) for coordinate logging. The automated CEJ/ALC (x, y) predictions were compared using directional offsets (Δx, Δy). Accuracy was quantified using root mean square error (RMSE): √[(Σ(Δx² + Δy²)/n)], with <0.5 mm deemed clinically acceptable. Intraclass correlation coefficient (ICC >0.90) confirmed reliability. Subgroup analyses stratified by region/tooth type were performed using analysis of variance (ANOVA). A random forest regression model was used to identify the predictors of bone loss. All computations in the statistical analyses were performed using SciPy version 1.14.1 (SciPy Project, open-source community-driven, coordinated by NumFOCUS, Austin, TX, USA), with p < 0.05.

## Results

The study cohort comprised 200 patients with nearly equal sex distribution, with 104 (52%) males and 96 (48%) females. The majority of patients were non-smokers. Alveolar bone loss, the key outcome variable, was observed in 124 patients (62%). The sample was balanced in terms of sex, thereby reducing the potential sex-based bias. However, the high prevalence of alveolar bone loss indicates notable periodontal involvement in this population (Table 3).

**Table 3:** Demographic characteristics of the categorical dataset. Data are presented as frequency (n) and percentage (%), where n denotes the number of patients.

Variables	Category	n (%)
Sex	Female	96 (48)
	Male	104 (52)
Smoking	No	120 (60)
	Yes	80 (40)
Alveolar bone loss	No	76 (38)
	Yes	124 (62)

The patients had a mean age of 23.6 ± 2.37 years. The average periodontal index was 1.90 ± 0.57 mm, and the mean duration of orthodontic treatment was 26.72 ± 4.41 months. The mean ALC level was 1.35 ± 0.65 mm in the anterior region and 1.47 ± 0.62 mm in the posterior region. The sample consisted of young adults who had undergone standard orthodontic treatment. The periodontal index value indicated a patient population with mild-to-moderate periodontal inflammation. The slightly greater bone loss observed in the posterior region than in the anterior region is a common clinical finding, possibly because of the differences in biomechanical forces and bone density (Table 4).

**Table 4:** Baseline characteristics of the continuous dataset in orthodontic patients. Data are presented as mean and standard deviation (SD), CEJ: cementoenamel junction, ABC: alveolar bone crest

Variables	Unit	Mean ± SD
Age	Years	23.6 ± 2.37
Periodontal index	mm	1.90 ± 0.57
Duration of treatment	Months	26.72 ± 4.41
Average height CEJ to ABC (anterior jaw)	mm	1.35 ± 0.65
Average height CEJ to ABC (posterior jaw)	mm	1.47 ± 0.62

The YOLOv8 model demonstrated high performance, with precision and recall scores of 0.943. The mAP at a CIoU threshold of 0.5 (mAP50) was 0.941, while the more stringent metric across CIoU thresholds from 0.5 to 0.9 (mAP50-95) was 0.805. The model exhibited excellent accuracy in detecting objects (teeth), as shown by its high precision and recall, indicating that it correctly identified nearly all relevant objects with very few false positives or false negatives. A high mAP50 confirmed robust performance under standard detection conditions. The decrease in mAP50-95 indicated that while detection was accurate, the precision of the bounding boxes' fit to the objects decreased under stricter localization criteria. The overall tooth detection accuracy achieved by the YOLOv8 model is 95.44%. This high accuracy rate confirmed that the model is highly effective and reliable for the primary task of identifying and localizing teeth in radiographic images. Successful detection is a crucial step that enables detailed segmentation and analysis (Table 5).

**Table 5:** YOLOv8 detection results. mAP: mean average precision

Model	Precision	Recall	mAP50	mAP50-90
YOLOv8	0.943	0.943	0.941	0.805

For the tooth mask, the accuracy improved from 91.43% to 92.28% after image augmentation. Similarly, the bone mask accuracy increased from 93.60% to 94.15%. In contrast, the crown mask accuracy decreased slightly from 95.19% to 94.11% after augmentation. Image augmentation is generally beneficial because it enhances the performance of the model for segmenting complex structures, such as teeth and bones, by increasing dataset variability and reducing overfitting. The crown mask, which achieved the highest baseline accuracy, was likely less dependent on augmentation for learning the distinctive features. The minor decrease in performance could be due to the introduction of unhelpful variations that slightly disrupted the learning of well-defined contours, suggesting that augmentation strategies should be tailored to specific anatomical structures (Table 6).

**Table 6:** Accuracy of mask region-based convolutional neural network (Mask R-CNN) model for dental structure segmentation with and without image augmentation. The data represent the accuracy (%) of the Mask R-CNN model for segmenting tooth, bone, and crown masks. Original refers to performance on unprocessed images, while image augmentation refers to performance on images enhanced with data augmentation techniques (such as rotation, flipping, or scaling).

Mask Category	Original	Image Augmentation
Tooth mask	91.43%	92.28%
Bone mask	93.60%	94.15%
Crown mask	95.19%	94.11%

**Figure 6:**
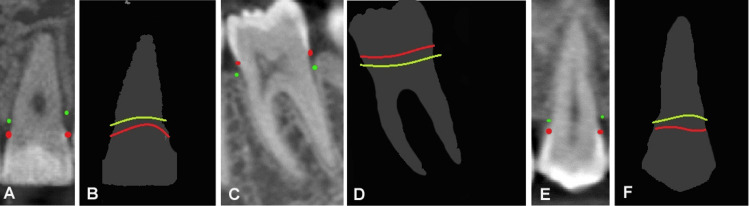
Comparison of cementoenamel junction (CEJ) and alveolar bone crest (ALC) positioning algorithm results with periodontist annotations (A) Periodontist annotation of CEJ (red points) and ALC (green points) on central incisor, (B) Algorithm annotation of CEJ (red line) and ALC (yellow line) on central incisor, (C) Periodontist annotation of CEJ (red points) and ALC (green points) on molar, (D) Algorithm annotation of CEJ (red line) and ALC (yellow line) on molar, (E) Periodontist annotation of CEJ (red points) and ALC (green points) on premolar, (F) Algorithm annotation of CEJ (red line) and ALC (yellow line) on premolar. Original images derived from the patient’s record.

The algorithm demonstrated high precision in locating the CEJ and ALC across different tooth types when compared to periodontist annotations. For CEJ detection, precision ranged from 85.3% to 94.5%, with the highest performance observed for right-sided molars (94.5%) and the lowest for right-sided premolars (85.3%). Similarly, ALC detection precision ranged from 82.8% to 95.3%, with right-sided molars showing the highest accuracy (95.3%) and left-sided premolars showing the lowest (82.8%). The RMSE values, which indicate the average deviation of the measurements, were consistently low. The most precise localization was for the right ALC (RMSE = 0.023), whereas the least precise localization was for the left ALC (RMSE = 0.071) (Table 7, Figure 6).

**Table 7:** Precision and RMSE of AI-based CEJ and ALC positioning compared to periodontist annotations. Data represent precision (%) and root mean square error (RMSE, in mm) for AI-based positioning of CEJ (cementoenamel junction) and ALC (alveolar bone crest) compared to periodontist annotations. Left and right refer to tooth sides or image perspectives (such as left vs. right side of the dental arch).

Precision	Incisor	Molar	Premolar	RMSE
CEJ (left)	91.2	92.6	89.2	0.061
CEJ (right)	93.4	94.5	85.3	0.038
ALC (left)	89.4	91.3	82.8	0.071
ALC (right)	91.3	95.3	89.2	0.023

The random forest regression model identified the relative importance of various features in predicting alveolar bone loss. Based on the mean decrease in accuracy, the duration of orthodontic treatment was the most influential predictor (0.321), followed by the periodontal index (0.171), age (0.142), sex (0.076), and smoking status (0.057). A similar hierarchy was observed using the mean dropout loss metric, with treatment duration again having the highest impact (0.595), followed by periodontal index (0.445), age (0.437), sex (0.367), and smoking (0.344). The results strongly indicate that the duration of orthodontic treatment is the foremost predictor of alveolar bone loss in this model, suggesting that longer treatment times are associated with greater bone level changes. The moderate influence of age implies that biological age may be a factor in the bone-remodeling response (Table 8).

**Table 8:** Feature importance in the regression random forest model for predicting alveolar bone loss. Data represent feature importance metrics for predictors in a regression random forest model for alveolar bone loss, mean decrease in accuracy (MDA) measures the reduction in model accuracy when a feature is removed, mean dropout loss (MDL) measures the increase in prediction error when a feature is excluded. Higher values indicate greater importance.

Feature Importance Metrics	Mean Decrease in Accuracy (MDA)	Mean Dropout Loss (MDL)
Duration of treatment (months)	0.321	0.595
Periodontal index (mm)	0.171	0.445
Age (years)	0.142	0.437
Smoking	0.057	0.344
Sex	0.076	0.367

**Figure 7:**
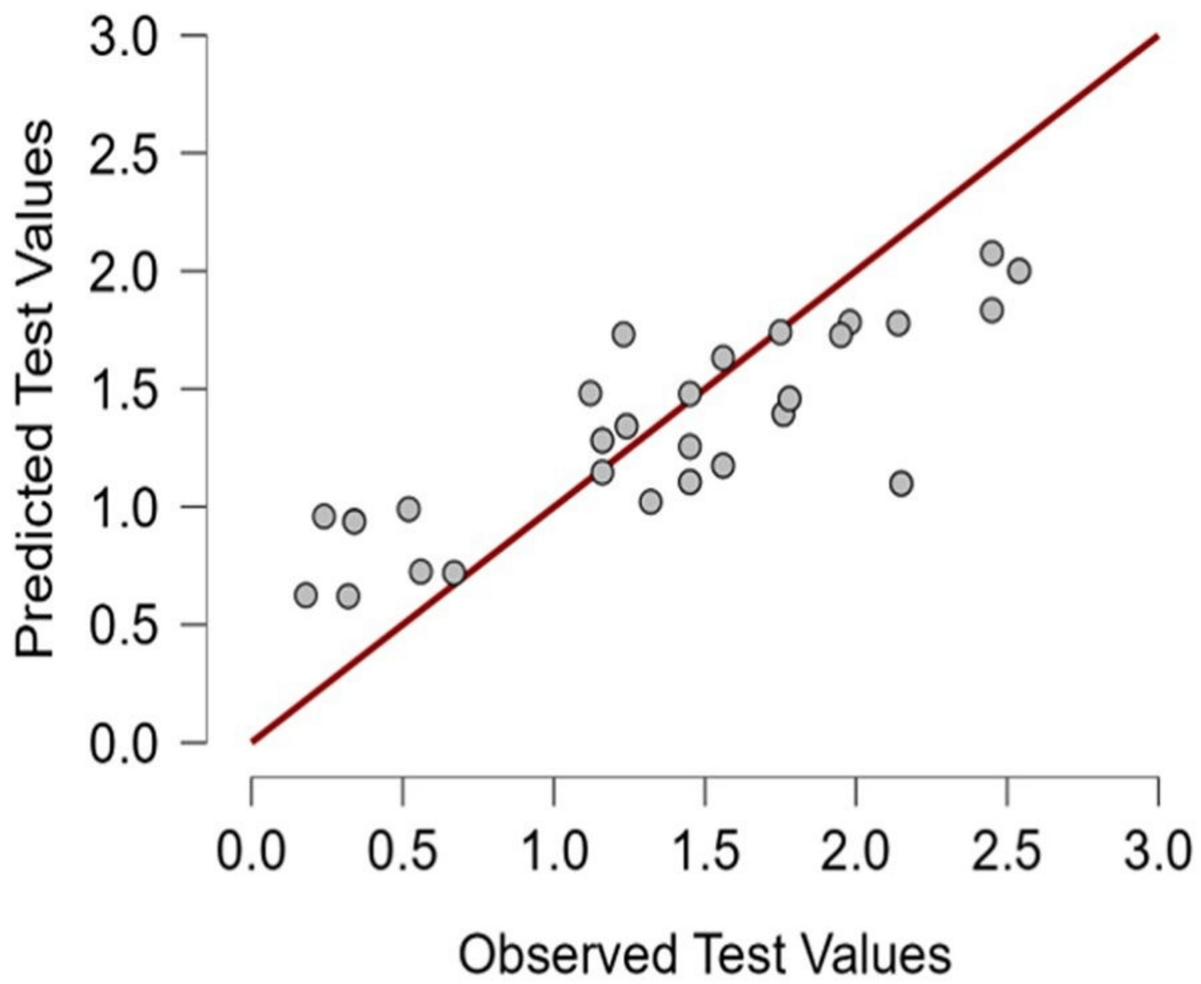
Predictive performance plot of random effect model analysis for alveolar bone loss. Gray dots: Individual data points comparing predicted and observed test values (in mm), Red line: Line of perfect prediction (where predicted values equal observed values).

The scatter plot shows the predictive performance of a regression random forest model for alveolar bone loss. The close alignment of the gray data points along the red line of perfect prediction suggests that the model has a strong ability to predict observed test values accurately. The spread of points around the line indicates some variability, but the overall trend shows good agreement between predicted and observed values, implying the model is reliable for this dataset (Figure 7).

## Discussion

The integration of AI methodologies in dental imaging, as demonstrated in this study, aligns with the emerging trends in automated segmentation and landmark detection, enhancing the precision of post-orthodontic periodontal assessments [6,7]. The high performance of the YOLOv8 model for tooth instance segmentation corroborates the findings of prior research, where deep learning architectures have achieved comparable accuracy in processing CBCT images [8]. For instance, a fully automatic AI system for tooth and alveolar bone segmentation reported robust results across real patient datasets, emphasizing the efficiency of single-stage detectors such as YOLO in handling complex anatomical variations [9]. Similarly, evolution in deep learning for tooth segmentation from CT/CBCT has shown mAP scores exceeding 0.90 under standard thresholds, supporting the observed mAP50 of 0.941 as indicative of reliable detection for downstream tasks [8]. The slight decline in mAP50-95 to 0.805 under stricter criteria can be due to the fact that bounding box precision diminishes in high-resolution images owing to subtle edge artifacts, yet the overall accuracy remains clinically viable. These benchmarks validate the effectiveness of the pipeline, positioning it as a scalable alternative to manual methods that are prone to interobserver variability.

The role of data augmentation in refining the segmentation accuracy for tooth and bone mask echoes established evidence that augmented datasets mitigate overfitting in dental AI applications [10]. Studies have shown that techniques such as flipping and rotation consistently elevate performance in segmenting maxillofacial structures, with improvements in accuracy for complex tissues [10,11]. The observed enhancement from 91.43% to 92.28% for tooth masks and from 93.60% to 94.15% for bone masks aligns with previous literature on deep learning segmentation, where augmentation introduces variability to better capture trabecular patterns in the alveolar bone [10,12]. The marginal dip in crown mask accuracy post-augmentation may reflect over-introduction of noise for well-defined enamel structures, a phenomenon reported in prior work where simpler features benefit less from aggressive transformations [12]. This selective impact underscores the need for tailored augmentation strategies, as observed in automatic tooth segmentation models that achieve high efficiency through balanced data enhancement [13].

The precision in localizing CEJ and ALC landmarks, particularly for molars, is substantiated by advanced AI systems that demonstrate over 95.94% accuracy in periodontal bone loss on CBCT images [7]. Research on AI for detecting alveolar bone loss using panoramic radiographs has reported a 97% accuracy, 90% sensitivity, 96% specificity, and an F1 score of 0.80 for total alveolar bone loss. This superior performance is attributed to the YOLOv8 model, which excels in delineating junctions with varying bone densities [14]. The F1 score was highest when estimating alveolar bone loss in the maxillary anterior region. Regional variations with better right-sided detection may stem from asymmetric training data distributions, a common insight from studies comparing AI to human annotations in panoramic and CBCT analyses [15,16].

The emphasis of the random forest model on orthodontic treatment duration as the primary predictor of alveolar bone loss is supported by previous studies linking prolonged force application to increased resorption [17,18]. Evidence indicates that treatments exceeding 24 months correlate with a 0.75 ± 19.85% reduction in bone density due to sustained biomechanical stress on periodontal ligaments [19]. The secondary influence of periodontal index and age aligns with research on age-related maladaptation, where older adults exhibit slower remodeling and heightened alveolar bone loss with orthodontic treatment [20]. The lesser roles of sex and smoking are consistent with a previous study identifying them as modifiable factors, although they are less dominant than duration in multivariate models [21,22]. These hierarchies reinforce the biological basis for bone changes and integrate the inflammatory and demographic variables.

Clinically, this AI pipeline offers transformative implications for periodontal monitoring after orthodontic treatment, enabling rapid, non-invasive assessments that could reduce the diagnostic time by 80% and facilitate early interventions to prevent progression to severe disease. Automating CEJ-ALC measurements supports personalized care and improves outcomes in aligner therapies and remote monitoring, as evidenced by AI's positive effects on hygiene and treatment adherence. Limitations include the small sample size and retrospective design, which may introduce selection bias and limit causal inferences, as confounding variables such as compliance were not controlled. The cohort's focus on young adults restricts generalizability to older populations, and AI's reliance on high-quality CBCT may falter with artifacts, highlighting the need for prospective validation and ethical data handling. The analysis was limited to vertical (CEJ-ALC) measurements, and buccal/lingual cortical bone thickness was not evaluated. Although CBCT allows such assessment, potential artifact interference and resolution variability could affect accuracy; future studies should incorporate these dimensions for more comprehensive orthodontic bone monitoring. Future studies will extend this AI framework to incorporate three-dimensional cortical bone thickness analysis and validation against expert clinical assessments for comprehensive orthodontic bone monitoring.

## Conclusions

This study successfully developed and validated an automated AI pipeline for evaluating alveolar bone levels in pre- and post-fixed orthodontic therapy patients by using CBCT imaging. By leveraging YOLOv8 for tooth instance segmentation and Mask R-CNN for precise localization of the CEJ and ALC, the pipeline demonstrated robust detection and segmentation capabilities. Data augmentation enhances the model performance for complex structures such as teeth and bone, whereas custom algorithms ensure accurate landmark localization across various tooth types. A random forest regression model highlighted treatment duration as a key predictor of alveolar bone loss. This AI-driven approach offers an efficient and scalable alternative to manual radiographic analysis, facilitating early detection of bone changes and personalized care. Future studies should pursue prospective validation and broader patient diversity in order to enhance clinical integration.
